# Pregnancy complications among nulliparous and multiparous women with advanced maternal age: a community-based prospective cohort study in China

**DOI:** 10.1186/s12884-020-03284-1

**Published:** 2020-10-02

**Authors:** Jiayou Luo, Chunli Fan, Miyang Luo, Junqun Fang, Shujin Zhou, Fenfang Zhang

**Affiliations:** 1 Department of Women and Children Health, School of Public Health, Central South University, Changsha, Hunan Province China; 2 Hunan Provincial Key Laboratory of Clinical Epidemiology, Changsha, Hunan Province China; 3 Department of Gynecology, Maternal and Child Health Hospital of Hubei Province, Wuhan, Hubei Province China; 4 Department of Child Health Care, Hunan Provincial Maternal and Child Health Care Hospital, Changsha, Hunan Province China; 5 Department of Health, Liuyang Maternal and Child Health Care Hospital, Liuyang, Hunan Province China

**Keywords:** Community-based prospective cohort, Advanced maternal age, Pregnancy complications, Incidence

## Abstract

**Background:**

This study aimed to evaluate the incidence rates and risks of pregnancy complications among nulliparous and multiparous women with advanced maternal age (AMA, ≥35 years) in China.

**Methods:**

We performed a community-based prospective cohort study of 10,171 pregnant women in selected two sub-districts and 11 towns of Liuyang from 2013 to 2015. All subjects were followed up from the first prenatal care (at ≤12 weeks) to delivery, and risks of pregnancy complications were compared by parity and maternal age groups.

**Results:**

Among nulliparas, women with AMA showed significantly increased risks for gestational hypertension (OR 8.44, 95%CI 1.68–2.88), preeclampsia/eclampsia (OR 9.92, 95%CI 4.87–18.78), premature rupture of membrane (OR 6.84, 95%CI 2.00–17.69), as compared to women in the 20–29-year age group. Among multiparas with AMA, increased risks were found for gestational diabetes mellitus (OR 3.29, 95%CI 1.76–5.94), anemia (OR 1.85, 95%CI 1.25–2.69), polyhydramnios (OR 3.29, 95%CI 1.56–6.64), premature rupture of membrane (OR 5.14, 95%CI 2.12–12.29), and preterm labor (OR 1.89, 95CI 1.42–2.50).

**Conclusions:**

Women with AMA were associated with increased risks of pregnancy complications, and complications with increased risks differed in nulliparas and multiparas. Women with AMA should be identified as a high-risk group in clinical practice.

## Background

Since the 1970s, the Chinese government has implemented the one-child policy to control the rapid growth of the population for around 40 years. With emerging problem of the aging population, the population policy has gradually switched to the two-child policy which encourages the birth of a second child since 2011 and has been fully implemented since 2016 [1]. With the impact of this new population policy, China is expected to see a sharp increase in fertility rate, and the proportion of women with advanced maternal age (AMA) is estimated to increase significantly [2]. Women with AMA may have increased risk for complications during pregnancy due to decreased ovarian and uterine function, as well as other concomitant diseases, which may result in threats to maternal and child health [3]. Thus, the prevention and control of complications in women with AMA during pregnancy have become a major challenge in clinical practice.

Previous studies have reported that women with AMA had higher incidences of pregnancy complications, such as hypertensive disorders of pregnancy, gestational hypertension, placental disorders, preterm labor, maternal near miss, and maternal death [4–7]. Moreover, research also suggested that the elevated risks of pregnancy complications in AMA may differ by parity [8], and inconsistent results were reported regarding certain complications [9–11]. For instance, some studies found that the risk of gestational diabetes and hypertensive disorders of pregnancy were increased in both nulliparous and multiparous women with AMA [9], while in some studies the increased risks for the two complications were not observed in both nulliparas and multiparas [10, 12]. It is therefore important to examine the association between AMA and pregnancy complications by parity within different populations and consider various confounding factors, such as disease history and lifestyle factors.

To our knowledge, only a few studies analyzed pregnancy complications and AMA in China previously, and these studies had certain limitations. The majority of previous studies were case-control or cross-sectional studies, and most studies did not conduct stratified analysis by parity [13, 14]. Although there were two retrospective cohort studies that analyzed pregnancy complications and AMA in China [15, 16], these two studies only recruited patients with pregnancy complications in hospitals, thus incidence rates of different pregnancy complications among different age groups were unknown. Also, previous studies mainly focused on the population lived in urban areas, while few studies focused on women lived in rural areas. These women usually have a low educational level and low socioeconomic status, who may need more support from the healthcare system on health education and prenatal care [17].

Our study aimed to evaluate the incidence rates and risks of pregnancy complications among women with different parity and maternal age groups through a community-based prospective cohort study. This study may highlight the need for prevention of pregnancy complications among women with AMA in rural areas.

## Methods

### Study population

Subjects for this study were recruited from multiple maternal and child healthcare centers in Liuyang city, Hunan Province, China from June 2013 to December 2015 [18]. Liuyang is a county-level city located in the central-south region of China. It has four sub-districts and 33 towns under its jurisdiction. In this study, we randomly selected two sub-districts and 11 towns in Liuyang city to represent the population from both urban and rural areas, and the recruitment was conducted in the maternal and child healthcare centers located in each selected sub-district/ town.

Pregnant women were recruited by their gynecologists during their regular prenatal care visits with the following inclusion criteria: a) received the first prenatal care at the recruited healthcare center; b) had no more than 12 weeks of gestation. Subjects were excluded if they a) had pregnancy complications before recruitment in the first trimester of the index pregnancy because it’s unclear whether the disease was started from this pregnancy or before this pregnancy; b) had an induced abortion during follow-up or did not have complete medical records from enrollment through delivery; c) had language communication barriers or were deaf; or d) refused to actively cooperate for this research.

Informed written consent was obtained from all participants. Ethical approval was obtained from the Ethics Committee of Xiangya School of Public Health, Central South University, and all research was performed in accordance with relevant guidelines/regulations.

### Follow-up and data collection

Baseline information was collected for all subjects by trained research gynecologists through a face-to-face interview. The questionnaire included socio-demographic characteristics (i.e. age, education, occupation, family income, residential area), pre-pregnancy weight and height, and history of gravidity, parity, and abortion. Routine obstetric examinations and laboratory examinations were also conducted afterward. Subjects were followed up at 16 weeks, 24 weeks, 28 weeks, 32 weeks, 38 weeks of pregnancy (or before delivery), and the end of delivery, and routine obstetric examinations were conducted at each follow-up. During the follow-up period, medical records including the records of routine obstetric care, the diagnosis of pregnancy complications, and the time of diagnosis were collected. A supervisory team was assigned to check whether the follow-up was on time, verify the registered complications, and check and rearrange the missing diagnosis.

### Variable definitions

Subjects were categorized into four maternal age groups, including 20–25 years, 25–29 years, 30–34 years, and ≥ 35 years. Women with AMA were defined as age greater or equal to 35 years old. Subjects aged below 20 years old were excluded in stratified analysis, due to small sample size (*n* = 101). Residential area was categorized into urban and rural residence. Family per capita annual income was classified into four categories (in Chinese Yuan, CNY): ≤10,000, 10,001–20,000, 20,001–30,000, and ≥ 30,001. Education levels were classified into two categories: junior high school or lower and high school/college. Occupations included farmer, housewife, factory worker, and others.

Pre-pregnancy body mass index (BMI) was calculated using the formula weight (kg)/height (m)^2^. BMI was categorized into three categories using Asian-specific cut-offs [19]: < 18.5 kg/m^2^, 18.5–23 kg/m^2^, 23–27.5 kg/m^2^, and ≥ 27.5 kg/m^2^. Gravidity and parity were categorized as none, once or more pregnancies/births. History of miscarriages was classified into three categories: none, once, twice and more times, and history of induced abortion and history of preterm labor was categorized as none and once or more times.

Gestational hypertension was defined as having high blood pressure after 20 weeks according to the International Society for the Study of Hypertension in Pregnancy guidelines [20]. Chronic hypertension before 20 weeks of gestation was not an outcome of interest in this study. Gestational diabetes mellitus (GDM) was diagnosed by oral glucose tolerance test (OGTT) conducted at 24–28th week of gestation, with fasting plasma glucose > 5.6 mmol/l or with post-75 g glucose load glucose level of > 8.8 mmol/l in 60th min or > 7.8 mmol/l in 120th min [21]. Anemia was defined as hemoglobin < 12.0 g/dL [22]. Polyhydramnios and oligohydramnios were examined using ultrasound, and was defined as the 4-quadrant amniotic fluid index (AFI) > 24 cm or ≤ 5 cm, respectively. Threatened abortion was defined as a small amount of vaginal bleeding, followed by paroxysmal lower abdominal pain or low back pain before the 28th week of pregnancy. The pelvic examination was not open, the membrane was intact, no pregnancy was discharged, and the size of the uterus was consistent with the gestational age. Preterm labor was defined as birth occurs before the start of the 37th week of pregnancy. Low birth weight is defined as the birth weight of an infant of 2499 g or less.

### Statistical analysis

The demographic and pre-pregnancy characteristics of study participants were compared among four maternal age groups. Significant levels were compared using Chi-square tests, and all the tests were two-tailed. Incidence of pregnancy complications and mode of delivery were compared by parity and age groups, and we used subjects age 20 to 29 years as the reference group considering the low incidence of pregnancy complications in lower age groups. Logistic regression was used to calculate the odds ratio (OR) and 95% confidence interval (CI) for pregnancy complications. Univariate analyses were conducted on all socio-demographic and pre-pregnancy characteristics to select the variable for adjustment, and significant variables were included in the multivariable model. Cramer’s V statistics were used to evaluate the correlation between variables included in the final model. We were not able to conduct logistic regression for placenta previa, infectious disease, and threatened abortion among nulliparas due to low incidence rate. In the multivariate analysis, income level and BMI were adjusted for gestational hypertension, preeclampsia/eclampsia, and threatened abortion; occupation was adjusted for GDM and premature rupture of membrane; income level was adjusted for anemia, polyhydramnios, oligohydramnios, placenta previa, and infectious disease; occupation and history of preterm labor was adjusted for preterm labor; education and BMI were adjusted for the mode of delivery and low birth weight. All analyses were conducted using R version 3.5.2.

## Results

### Socio-demographic and pre-pregnancy characteristics

**Fig. 1:**
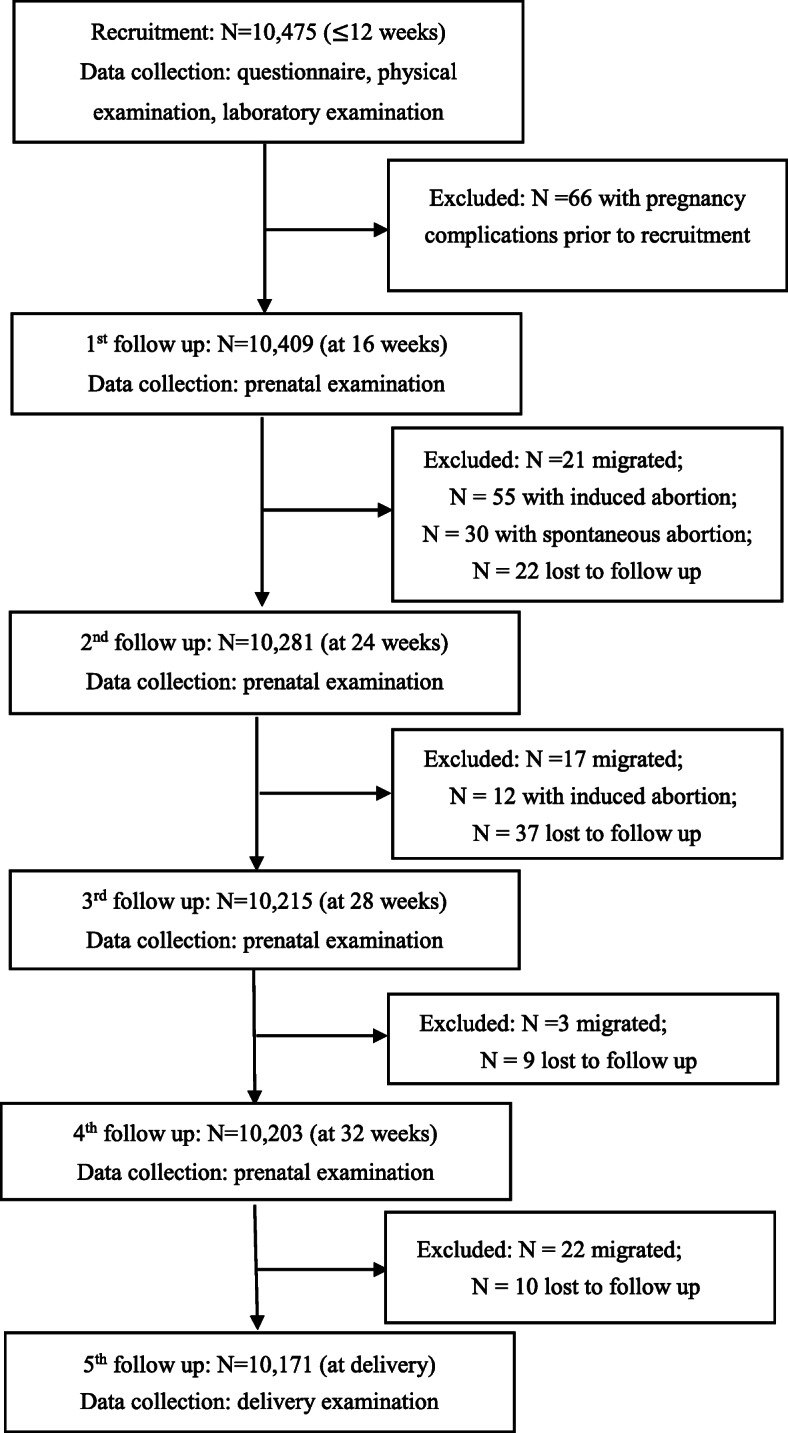
Flow of inclusion to the current analysis

A total of 12,170 pregnant women were admitted to the hospital from June 2013 to December 2015. Among them, 10,475 pregnant women were recruited in the study (response rate 86.1%), and 10,171 subjects with complete information were included in the final analysis (97.1%) (Fig. 1).

Among the 10,171 subjects, the average age was 26.7 ± 4.2 years. 3274 (32.2%) subjects were 20–24 years old, 4587 (45.1%) subjects were 25–29 years old, 1637 (16.1%) subjects were 30–34 years old, and 572 (5.6%) subjects were 35 years old and older (Table 1). The majority of subjects (85.9%) lived in rural areas, and the median family annual income was 20,000 Chinese Yuan per capita (around 2880 USD). The majority of subjects did not complete high school education (83.7%), and most of them were housewives (54.3%), factory workers (23.5%), and farmers (13.9%). The pre-pregnancy BMI was normal for 61.4% of subjects, 18.0% of subjects were underweight, and 20.5% of subjects were overweight/obese. Overall, around half of the subjects had one or more births previously, and the proportions were much higher for women aged 30–35 years (79.0%) and women aged 35 years and above (88.6%) than younger age groups. Also, around one-third of women aged 35 years and above had two or more times of miscarriages, while the proportion ranged from 6.3 to 19.5% in younger age groups. 3.1% of subjects had a history of induced abortion, and the proportion ranged from 1.7 to 5.9% in different age groups.

**Table 1:** Socio-demographic and pre-pregnancy characteristics of study participants

Variables	Overall	Maternal age groups (years)				*p*
		20–24	25–29	30–34	≥35	
(n)	(10,171)	(3274)	(4587)	(1637)	(572)	
Residential area (n, %)						< 0.001
Urban	1437 (14.1)	396 (12.1)	742 (16.2)	222 (13.6)	68 (11.9)	
Rural	8734 (85.9)	2878 (87.9)	3845 (83.8)	1415 (86.4)	504 (88.1)	
Family per capita annual income (Chinese Yuan, n, %)						0.004
≤ 10,000	1647 (16.2)	550 (16.8)	684 (14.9)	267 (16.3)	124 (21.7)	
10,001–20,000	4147 (40.8)	1409 (43.0)	1805 (39.4)	660 (40.3)	223 (39.0)	
20,001–30,000	2871 (28.2)	922 (28.2)	1345 (29.3)	442 (27.0)	141 (24.7)	
≥ 30,001	1506 (14.8)	393 (12.0)	753 (16.4)	268 (16.4)	84 (14.7)	
Education level (n, %)						< 0.001
Junior high school or lower	8511 (83.7)	2822 (86.2)	3604 (78.6)	1456 (88.9)	532 (93.0)	
High school/college	1660 (16.3)	452 (13.8)	983 (21.4)	181 (11.1)	40 (7.0)	
Occupation (n, %)						< 0.001
Farmer	1413 (13.9)	398 (12.2)	582 (12.7)	285 (17.4)	134 (23.4)	
Housewife	5521 (54.3)	1768 (54.0)	2447 (53.3)	924 (56.4)	317 (55.4)	
Factory worker	2395 (23.5)	819 (25.0)	1121 (24.4)	339 (20.7)	97 (17.0)	
Other	842 (8.3)	289 (8.8)	437 (9.5)	89 (5.4)	24 (4.2)	
Pre-pregnancy BMI (kg/m^2^, n, %)						< 0.001
< 18.5	1834 (18.0)	713 (21.8)	836 (18.2)	205 (12.5)	50 (8.7)	
18.5–23	6247 (61.4)	2028 (61.9)	2854 (62.2)	994 (60.7)	317 (55.4)	
23–27.5	1792 (17.6)	461 (14.1)	764 (16.7)	382 (23.3)	168 (29.4)	
≥ 27.5	298 (2.9)	72 (2.2)	133 (2.9)	56 (3.4)	37 (6.5)	
Gravidity (n, %)						< 0.001
None	3878 (38.1)	1861 (56.8)	1708 (37.2)	203 (12.4)	26 (4.5)	
Once or more pregnancies	6293 (61.9)	1413 (43.2)	2879 (62.8)	1434 (87.6)	546 (95.5)	
Parity (n, %)						< 0.001
None	5354 (52.6)	2445 (74.7)	2406 (52.5)	344 (21.0)	65 (11.4)	
Once or more births	4817 (47.4)	829 (25.3)	2181 (47.5)	1293 (79.0)	507 (88.6)	
History of miscarriage (n, %)						< 0.001
None	6903 (67.9)	2506 (76.5)	3129 (68.2)	927 (56.6)	256 (44.8)	
Once	1988 (19.5)	563 (17.2)	891 (19.4)	390 (23.8)	130 (22.7)	
Twice or more times	1280 (12.6)	205 (6.3)	567 (12.4)	320 (19.5)	186 (32.5)	
History of induced abortion (n, %)						< 0.001
None	9854 (96.9)	3218 (98.3)	4451 (97.0)	1546 (94.4)	538 (94.1)	
Once or more times	317 (3.1)	56 (1.7)	136 (3.0)	91 (5.6)	34 (5.9)	
History of preterm labor (n, %)						0.007
None	10,110 (99.4)	3262 (99.6)	4562 (99.5)	1620 (99.0)	565 (98.8)	
Once or more times	61 (0.6)	12 (0.4)	25 (0.5)	17 (1.0)	7 (1.2)	

### Incidence rates of pregnancy complications by parity and maternal age groups

The comparison of incidence rates for different pregnancy complications stratified by parity and age groups is shown in Table 2. Overall, the incidence rate of each complication during pregnancy ranged from 0.9 to 4.2%, and the top three pregnancy complications observed included gestational hypertension (4.2%), preeclampsia or eclampsia (3.5%), and anemia (3.7%). The incidence of pregnancy complications showed an increasing pattern when stratified by age groups for gestational hypertension, preeclampsia, polyhydramnios, premature rupture of membrane among nulliparas, and this pattern was observed for GDM, anemia, polyhydramnios, oligohydramnios, placenta previa, premature rupture of membrane among multiparas. The incidence of preterm labor was 9.4%, which ranged from 9.3 to 13.8% and 8.1 to 14.5% among different age groups in nulliparas and multiparas, respectively. The incidence of low birth weight was 2.8%, and the incidence was higher in multiparas (5.2%) than nulliparas (0.7%). In terms of mode of delivery, around half of subjects aged 30 years old and above performed a cesarean delivery, while this proportion was around 30% in the 20–29-year age group.

**Table 2:** Incidences of pregnancy complications by parity and maternal age groups

Variables	Overall	Nulliparas				Multiparas			
		20–29y^a^	30–34y	≥35y	*p*	20–29y	30–34y	≥35y	*p*
(n)	(10,171)	(4851)	(344)	(65)		(3010)	(1293)	(507)	
Gestational hypertension (n, %)	430 (4.2)	126 (2.6)	34 (9.9)	12 (18.5)	< 0.001	125 (4.2)	109 (8.4)	20 (3.9)	< 0.001
Preeclampsia/eclampsia (n, %)	352 (3.5)	106 (2.2)	167 (4.9)	12 (18.5)	< 0.001	114 (3.8)	87 (6.7)	16 (3.2)	< 0.001
Gestational diabetes mellitus (GDM, n, %)	145 (1.4)	63 (1.3)	10 (2.9)	1 (1.5)	0.092	31 (1.0)	23 (1.8)	17 (3.4)	< 0.001
Anemia (n, %)	380 (3.7)	151 (3.1)	6 (1.7)	6 (9.2)	0.006	119 (4.0)	59 (4.6)	37 (7.3)	0.004
Polyhydramnios (n, %)	89 (0.9)	32 (0.7)	4 (1.2)	3 (4.6)	0.001	21 (0.7)	17 (1.3)	12 (2.4)	0.001
Oligohydramnios (n, %)	101 (1.0)	25 (0.5)	13 (3.8)	1 (1.5)	< 0.001	36 (1.2)	17 (1.3)	8 (1.6)	0.462
Placenta previa (n, %)	146 (1.4)	33 (0.7)	10 (2.9)	0 (0.0)	< 0.001	52 (1.7)	37 (2.9)	14 (2.8)	0.086
Premature rupture of membrane (n, %)	90 (0.9)	43 (0.9)	7 (2.0)	4 (6.2)	< 0.001	11 (0.4)	14 (1.1)	10 (2.0)	< 0.001
Infectious disease (n, %)	158 (1.6)	29 (0.6)	3 (0.9)	0 (0.0)	< 0.001	75 (2.5)	36 (2.8)	14 (2.8)	< 0.001
Threatened abortion (n, %)	192 (1.9)	59 (1.2)	0 (0.0)	0 (0.0)	0.154	76 (2.5)	48 (3.7)	9 (1.8)	< 0.001
Preterm labor (n, %)	950 (9.4)	450 (9.3)	38 (11.1)	9 (13.8)	0.37	241 (8.1)	130 (10.1)	73 (14.5)	< 0.001
Low birth weight (n, %)	289 (2.8)	37 (0.8)	1 (0.3)	1 (1.5)	0.004	178 (5.9)	45 (3.5)	25 (4.9)	0.002
Mode of delivery (n, %)					< 0.001				< 0.001
Vaginal birth	6371 (64.5)	3401 (72.0)	189 (56.9)	94 (62.3)		1852 (63.1)	592 (47.4)	243 (49.6)	
Caesarean	3506 (35.5)	1321 (28.0)	143 (43.1)	40 (63.5)		1081 (36.9)	657 (52.6)	247 (51.1)	

^a^Women aged 20–24 years and 25–29 years were combined as one age group considering the low incidence of pregnancy complications in the two groups

### ORs for pregnancy complications in different maternal age groups by parity

Adjusted ORs for pregnancy complications calculated using logistic regression models are shown in Table 3. The risks for gestational hypertension (OR 8.44, 95%CI 4.18–15.58) and preeclampsia/eclampsia (OR 9.92, 95%CI 4.87–18.78) were significantly higher for women with AMA above among nulliparas compared with women aged 20–29 years, while this association was not observed among multiparas. Among multiparas, women with AMA were found to have increased risks for GDM (OR 3.29, 95%CI 1.76–5.94), anemia (OR 1.85, 95%CI 1.25–2.69), polyhydramnios (OR 3.29, 95%CI 1.56–6.64), premature rupture of membrane (OR 5.14, 95%CI 2.12–12.29), preterm labor (OR 1.89, 95%CI 1.42–2.50), while similar associations were only observed for premature rupture of membrane (OR 6.84, 95%CI 2.00–17.69) among nulliparas. Odds of performing cesarean delivery over virginal birth were significantly higher for women in older age groups as compared to the reference group both among nulliparas and multiparas.

**Table 3:** Multivariable adjusted odds ratios (ORs) for pregnancy complications by parity and maternal age groups

Variables	Nulliparas		Multiparas	
	30–34y (OR, 95% CI)	≥35y (OR, 95% CI)	30–34y (OR, 95% CI)	≥35y (OR, 95% CI)
Gestational hypertension^a^	4.17 (2.76–6.14)*	8.44 (4.18–15.85)*	2.2 (1.68–2.88)*	0.97 (0.58–1.54)
Preeclampsia/eclampsia^a^	2.39 (1.36–3.94)*	9.92 (4.87–18.78)*	1.91 (1.43–2.55)*	0.86 (0.48–1.42)
Gestational diabetesmellitus (GDM) ^b^	2.22 (1.06–4.18)	1.08 (0.06–5.04)	1.74 (1.00–2.99)	3.29 (1.76–5.94)*
Anemia^c^	0.57 (0.22–1.18)	3.13 (1.19–6.84)	1.15 (0.83–1.57)	1.85 (1.25–2.69)*
Polyhydramnios^c^	1.81 (0.54–4.62)	6.8 (1.60–19.82)	1.86 (0.96–3.53)	3.29 (1.56–6.64)*
Oligohydramnios^c^	7.59 (3.73–14.74)*	3.03 (0.17–14.67)	1.11 (0.61–1.96)	1.36 (0.59–2.81)
Placenta previa^c^	–	–	1.67 (1.08–2.54)	1.6 (0.84–2.82)
Premature rupture of membrane^b^	2.29 (0.93–4.82)	6.84 (2.00–17.69)*	2.89 (1.31–6.54)	5.14 (2.12–12.29)*
Infectious disease^c^	–	–	1.09 (0.72–1.62)	1.08 (0.58–1.86)
Threatened abortion^a^	–	–	1.45 (0.99–2.09)	0.66 (0.31–1.27)
Preterm labor^d^	1.22 (0.85–1.71)	1.54 (0.7–2.99)	1.27 (1.01–1.59)	1.89 (1.42–2.50)*
Low birth weight^e^	0.38 (0.02–1.77)	1.86 (0.1–8.93)	0.55 (0.39–0.77)*	0.78 (0.49–1.17)
Caesarean^e^ (ref. vaginal birth)	1.88 (1.5–2.36)*	4.06 (2.43–6.93)*	1.84 (1.61–2.11)*	1.65 (1.36–2.01)*

Women aged 20–29 years were used as the reference group. * Indicate *p*-value was significant after Bonferroni correction

^a^adjusted for annual income and BMI; ^b^adjusted for occupation; ^c^adjusted for income; ^d^adjusted for occupation and history of preterm labor; ^e^ adjusted for education and BMI

## Discussion

In this study, we found that women with AMA had a significantly higher incidence of pregnancy complications than women with younger age, and the increased risks of pregnancy complications varied by parity. More specifically, nulliparous women with AMA showed increased risks for gestational hypertension, preeclampsia/eclampsia, and premature rupture of membrane, while multiparous women with AMA showed increased risks for GDM, anemia, polyhydramnios, premature rupture of membrane, and preterm labor. Cesarean delivery was also associated with older maternal age, where increased risks were found for women aged 30–35 years and 35 years and above in both nulliparas and multiparas compared to women aged 20–29 years.

We found that increased risks of gestational hypertension and preeclampsia/eclampsia for AMA were only observed in nulliparous women, not in multiparous women. Our finding is consistent with many studies that reported preeclampsia was more common in nulliparas [23], while inconsistent with some previous studies that found AMA was a risk factor for hypertensive disorders during pregnancy in both nulliparous and multiparous women [9]. On the other hand, one study also suggested that AMA was not associated with preeclampsia irrespective of parity [12]. Our findings may be explained by several factors. First, studies have suggested that hypertensive disease in nulliparous and multiparous pregnant women may involve different pathophysiology, especially in terms of immune maladaptation, though no conclusion has been made so far [23, 24]. Moreover, it’s possible that the increased risk for the multiparous women with AMA was confounded by other risk factors, including history of hypertensive disorders in the first pregnancy, co-existence of other complications, and unhealthy lifestyle [25–27]. Future studies may be needed to examine the potential confounding factors, as well as the pathophysiology of pregnancy complications in women with different parity, to explore reasons behind this phenomenon.

Consistent with previous studies, the risks of GDM, anemia, polyhydramnios were increased in women with AMA compared to women aged 20–29 years in the multiparous group [7, 28]. GDM is a common pregnancy complication for women with AMA, and most scholars believe that the increased incidence may be due to change in blood volume, vascular endothelial injury, insulin receptor and insulin affinity decreased with aging [29–31]. Also, it has been reported that multiparity was associated with an increased risk of GDM [32], although the effects of increasing parity on insulin sensitivity or β-cell function were not detected [33]. Anemia during pregnancy is usually related to inadequate diets or not receiving prenatal iron and folate supplements [34]. Previous studies also reported that anemia was an independent risk factor for low birth weight and preterm delivery [35]. Considering the prevalence of underweight and low education level in this area, it’s important to provide health education and support to prevent and control anemia especially for women with AMA in healthcare practice.

A national survey reported that the overall cesarean delivery rate in China had increased from 28.8% in 2008 to 34.9% in 2014, with the rates increased linearly in rural and general urban areas but declined beyond baseline rates in super cities [36]. In this study, the overall cesarean delivery rate was 35.5%, and AMA was found to be a risk factor for cesarean delivery, which is consistent with previous research [37]. With the change in child policy, the health care system should be prepared to cope with the increasing challenge of women with AMA and history of cesarean delivery.

This study has some limitations. First, subjects in this study were recruited at about 12 weeks of gestation based on the regulation of pregnant women in the health management registration system, thus we might underestimate the incidence rates of complications during early pregnancy. Also, it is possible that some subjects did not participate in all of the screening tests suggested by their gynecologist, especially for those with low education and income level, which may result in a lower incidence rate. However, the results of our study reflected the incidence of pregnancy complications in routine clinical practice. Second, only a few subjects in extreme age groups were included in the study, thus we were unable to conduct stratified analysis for subjects aged younger than 20 years old and subjects aged 40 years and above. Third, the results may be impacted by unmeasured confounding factors as we were not able to adjust for pre-pregnancy lifestyle and behaviors such as diet, smoking, and physical activity, although we have conducted adjusted models for sociodemographic factors and pre-pregnancy BMI.

## Conclusions

To our knowledge, this is the first study to identify incidences of pregnancy complications in nulliparous and multiparous Chinese women with AMA using a community-based prospective cohort study. Our findings suggest that women with AMA should be regarded as high-risk groups for pregnancy complications in the practice of maternal health care and maternal care management. Also, different risks of pregnancy complications were observed for nulliparous and multiparous women with AMA. This study bears importance in early prevention of pregnancy complications in the practice of population-based pregnancy health care.

## Supplementary information

**Additional file 1.**

## Data Availability

The dataset generated and/or analyzed during the current study are not publicly available, but are available from the corresponding author on reasonable request.

## Abbreviations

AMA: Advanced maternal age
BMI: Body mass index
CNY: Chinese Yuan
GDM: Gestational diabetes mellitus
OGTT: Oral glucose tolerance test
