# Supplementary material for: Pregnancy complications among nulliparous and multiparous women with advanced maternal age: a community-based prospective cohort study in China
Source: BMC Pregnancy Childbirth. 2020 Oct 2;20:581. doi: 10.1186/s12884-020-03284-1 (PMC7532564; doi:10.1186/s12884-020-03284-1)
Supplement: Supplementary file 1 — Additional file 1. [file 12884_2020_3284_MOESM1_ESM.pdf]

Baseline questionnaire

Basic information

1. Name _______________ Ethnicity ___________ Age _____ years
2. Residential type: a) Urban b) Rural
3. Average household income ________________(Chinese Yuan)
4. Education level:
   a) Primary school or lower
   b) Junior high school
   c) High school/vocational school
   d) Collage/university
5. Occupation:
   a) Farmer
   b) Housewife
   c) Work in the service industry/run a business/company employee
   d) Factory worker
   e) Others: ______________
6. Pre-pregnancy height: ___________cm; pre-pregnancy weight: ____________kg

Past obstetric history

1. Gravidity (number of times you have been pregnant, regardless of the outcome): ______times
   Parity (total number of times you have given birth to a child with a gestational age of 24 weeks or more, regardless of whether the child was born alive or not):______ times
   History of preterm labor:______times
   History of stillbirth:______times
   History of miscarriage:______times
   History of induced abortion:______times
2. Date of the last miscarriage: ______________
3. Number of children alive: boys:____; girls:____
   Number of children with birth defects:_____
4. History of previous pregnancy complications: a) No b)Yes:________
